# The Effect of Foraging on Bumble Bees, *Bombus terrestris*, Reared under Laboratory Conditions

**DOI:** 10.3390/insects11050321

**Published:** 2020-05-23

**Authors:** Pavel Dobeš, Martin Kunc, Jana Hurychová, Alena Votavová, Olga Komzáková, Pavel Hyršl

**Affiliations:** 1 Department of Experimental Biology, Faculty of Science, Masaryk University, Kotlarska 2, 611 37 Brno, Czech Republic; jana.hurychova@mail.muni.cz (J.H.); hyrsl@sci.muni.cz (P.H.); 2 Research Institute for Fodder Crops, Ltd. Troubsko, Zahradni 1, 664 41 Troubsko, Czech Republic; 376041@mail.muni.cz (M.K.); votavova@vupt.cz (A.V.); olga.komzakova@mendelu.cz (O.K.)

**Keywords:** bumble bee, foraging, laboratory rearing, nutrients, immunity, physiology

## Abstract

Bumble bees are important pollinators broadly used by farmers in greenhouses and under conditions in which honeybee pollination is limited. As such, bumble bees are increasingly being reared for commercial purposes, which brings into question whether individuals reared under laboratory conditions are fully capable of physiological adaptation to field conditions. To understand the changes in bumble bee organism caused by foraging, we compared the fundamental physiological and immunological parameters of *Bombus terrestris* workers reared under constant optimal laboratory conditions with workers from sister colonies that were allowed to forage for two weeks in the field. Nutritional status and immune response were further determined in wild foragers of *B.* *terrestris* that lived under the constant influence of natural stressors. Both wild and laboratory-reared workers subjected to the field conditions had a lower protein concentration in the hemolymph and increased antimicrobial activity, the detection of which was limited in the non-foragers. However, in most of the tested parameters, specifically the level of carbohydrates, antioxidants, total hemocyte concentration in the hemolymph and melanization response, we did not observe any significant differences between bumble bee workers produced in the laboratory and wild animals, nor between foragers and non-foragers. Our results show that bumble bees reared under laboratory conditions can mount a sufficient immune response to potential pathogens and cope with differential food availability in the field, similarly to the wild bumble bee workers.

## 1. Introduction

Bumble bees are important pollinators of many agricultural crops. Their ability to adapt to the limited space of greenhouses and plant-breeding cages is nowadays widely utilized in the production of dozens of crops, e.g., tomatoes, peppers, cucumbers, soft fruits, stone fruits and seeds [1]. Furthermore, bumble bees are commercially produced for pollination in greenhouses and as starting nests they are provided to gardeners, who use them to support a population of pollinators also in urban areas. Although some populations of bumble bee species are in decline in many regions of the world [2,3], a commercial application of bumble bees should respect the natural species distribution to avoid possible disturbances in local ecosystems. Several species of bumble bee are already being reared in commercial hives, but only *Bombus terrestris* subspp. are used in the European region. The buff-tailed bumblebee *B. terrestris* is a eusocial insect that lives in colonies inhabited by several hundred individuals. Unlike honey bee workers, which initially act as nurses in the hive, bumble bee workers start to fulfill their duties both inside and outside of the nest a few days after hatching [4], reflecting the disparities in physiological development and senescence of the worker caste between both species. As a floral generalist, *B. terrestris* collects pollen and nectar from a broad spectrum of plant species which, along with its relatively easy commercial rearing, is the main reason for its wide use as a domesticated pollinator [1,5].

Pollen provides bumble bees with proteins, lipids and micronutrients, whereas floral nectar is the main source of carbohydrates and provides some essential amino acids [6,7,8]. When foraging, workers follow a dietary intake target, i.e., nutritional optimum, which is determined by age, reproductive capacity and physiological state of both individual workers and the colony [9,10,11,12]. A nutritional imbalance together with stress caused by chemical pesticides and immunological challenges are considered to be the main factors inflicting bee colony collapses [13]. Negative environmental conditions impact all levels of colony development and can exert rapid detrimental effects on bee foragers that are directly exposed to potentially harmful agents, such as insecticides. Furthermore, external factors can affect brood development in the nest when, for example, a brood pathogen is introduced to the colony through the foraging worker caste. Although bumble bees are able to forage in large areas and thus partially regulate unfavorable conditions, the distribution of floral sources and potential stress factors should still be carefully evaluated when selecting a locality for bumble bee field application.

So far, only a limited number of studies have focused on insect immune response during foraging [14,15,16,17]. Therefore, knowledge regarding changes in the immunity of laboratory-reared insects after their application in the field is still scarce. As in other insect species, innate immunity in bumble bees consists of cellular and humoral reactions. Several types of hemocytes are present in bumble bee hemolymph, in which they mediate phagocytosis, nodulation, encapsulation and contribute to coagulation [18]. Humoral response primarily includes the expression of genes coding for antimicrobial peptides and lectins, production of cytotoxic molecules such as reactive oxygen species, the phenoloxidase (PO) system and protein components of clotting cascade responsible for coagulation [19]. A trade-off between immunity and other traits, e.g., survival, reproduction or nutrient intake, has been the subject of numerous studies [20,21,22,23,24]; whereas foraging activity, which is costly due to high energy consumption during flight and sets high demands on the immune system, is only partially described in terms of fundamental physiological and immunological changes in the organism [14,15,17,25,26].

The aim of the current study was to address one of the main questions frequently asked by the public and farmers regarding the field application of animals produced in captivity, specifically, whether a commercially produced bumble bee species reared under laboratory conditions can adequately react to environmental stressors when applied in the field. The principal stress factors an organism must cope with when transferred from the laboratory to the field include a change in food accessibility and increased pathogen pressure, reflected in physiological and immunological changes, respectively. Specifically, we assumed that the foraging activity will cause a decrease in macronutrients circulating in the hemolymph, because of the high energy demands of flight [27], and will lead to a general weakening of defense reactions due to a trade-off with immunity [14,15]. To evaluate the possible effects of laboratory rearing, we subjected six sister colonies of *B. terrestris* to different lifestyle conditions and compared their physiological and immunological parameters with those of wild bumble bees by comparing their hemolymph properties. The list of tested parameters was selected to cover all fundamental components of the bumble bee immune system (i.e., hemocytes as the mediators of cellular immunity, antimicrobial activity, antioxidant capacity and phenoloxidase activity) and characterize their physiological status.

## 2. Materials and Methods

### 2.1. Bumble Bees

Our experiment was performed with six colonies of *Bombus terrestris* subsp. *terrestris* (Buff-tailed bumble bee) founded and led by sister queens to reduce the variability resulting from different genetic backgrounds. All colonies were produced at Agricultural Research, Ltd. Troubsko, Czech Republic and kept under the standard conditions prior to the experiment: i.e., dark, 25 °C and 55% relative humidity. They were provided with Apiinvert^®^ ad libitum and rapeseed pollen which was changed every two days. The colonies were kept in cages 27 × 23 × 16 cm (L × W × H) and all manipulations were performed under red light to minimize disturbance. When the colonies reached the size of typical nest intended for field application which contains at least 15–20 worker bees, they were randomly divided into two groups. Three colonies were kept henceforward under the standard rearing conditions without the opportunity to forage (control group). Remaining three colonies were provided heat insulation and applied in the field, specifically, on a shady place in a garden area in the surroundings of Troubsko, Czech Republic (GPS 49.174° N, 16.507° E), without any restrictions on foraging (forager group). We ensured that all colonies were at the same level of development and did not show any signs of disease or disease-related behavior during the experiment. All colonies were left for two weeks without any treatment except the feeding of control groups which continued as mentioned above. The experiment was conducted at the beginning of July; therefore, we assume that foraging bumble bees did not suffer from starvation or cold temperatures (the average temperature in July 2019 was 20.3 ± 3.0 °C) during the field application. The pollen and nectar sources available during the time of our study in the range of foraging bees include for instance *Trifolium* spp., *Centaurea* spp., *Vicia* spp., *Plantago* spp., *Lamium* spp., *Sinapis* spp., *Rosa* spp., *Lonicera* spp., *Tilia* spp. or *Robinia* spp. Wild workers of *B. terrestris* used in our study (wild forager group) were collected in the park area of Brno, Czech Republic (GPS 49.181° N, 16.570° E) in June 2019. This locality is roughly 5 km from agricultural areas and greenhouses where commercial bumble bee colonies are being applied and was selected to minimize a risk of unintentional sampling of bumble bees produced in captivity. It was not possible to determine the exact age of wild bumble bees, however only the individuals that did not show any visible signs of deformities or disease were used in the experiments. We suppose that the wild bumble bees used in our study were not older than 1.5 months, since in the middle European climatic conditions the wild nests of *Bombus terrestris* are usually established in late March and April [28,29].

### 2.2. Collection of Hemolymph Samples

Bumble bee workers were collected from the experimental colonies, anesthetized with CO_2_ and their abdomen was separated by scissors. The thorax was gently compressed; and the hemolymph was collected by calibrated pipette from a drop which appeared at the site of the wound. Approximately 6 µL was collected per individual bumble bee. Collected hemolymph was used immediately to determinate protein concentration, hemocyte counts and phenoloxidase activity, or frozen at −80 °C for other analysis. To protect the frozen samples from melanization, 1 µL of phenylthiourea (1 mg/mL; in phosphate buffer, pH 7.4) per each 5 µL of hemolymph was added to the samples. The hemolymph of the wild bumble bees was sampled as mentioned above and compared to the bumble bees reared in captivity.

### 2.3. Determination of the Total Level of Nutrients

Macronutrients, i.e., carbohydrates, lipids and proteins, were measured spectrophotometrically as their total pool in the hemolymph. For determination of carbohydrate level, the anthrone assay was used as described previously [30]. Briefly, the hemolymph samples stored with phenylthiourea at −80 °C were diluted 200× in PBS (pH 7.4), incubated with anthrone reagent (Sigma-Aldrich, St. Louis, MO, USA) and the resulting products were measured as absorbance at 620 nm using spectrophotometer Sense (Hidex, Turku, Finland). The concentration of carbohydrates was calculated according to calibration curve, which was prepared from a serial dilution of glucose (Sigma-Aldrich, St. Louis, MO, USA) and measured in parallel with the hemolymph samples. The lipid concentration was determined by the sulpho-phosho-vanillin method according to Zollner et al. [31] modified by Kodrík et al. [32] in 1 µL of the hemolymph which was stored with phenylthiourea at −80 °C before analysis. The reaction resulted in a change of absorbance which was measured at 546 nm by spectrophotometer Sunrise (Tecan, Männedorf, Switzerland) and converted to the lipid concentration according to calibration data acquired from a series of diluted oleic acid. The protein concentration was measured separately in each bumble bee individual using a commercial kit (Bio-Rad, Hercules, CA, USA). The measurement was performed immediately after the hemolymph collection without anticoagulant because phenylthiourea negatively affects the resulting absorbance. The absorbance was detected at 700 nm using spectrophotometer Multiscan GO (Thermofisher scientific, Waltham, MA, USA). The calibration curve was prepared from several dilutions of bovine serum albumin.

### 2.4. Counting of the Hemocytes

The circulating hemocytes were counted in hemocytometer under CX31 light microscope (Olympus, Tokyo, Japan) with phase contrast. To determine the hemocyte count, 10 µL of the hemolymph was sampled from five bumble bees (2 µL per individual) and mixed with 2.5 µL of phenylthiourea in phosphate buffer (1 mg/mL) to prevent melanization. The concentration of hemocytes was expressed as the hemocyte count per µL of the hemolymph.

### 2.5. Determination of the Antioxidant Capacity

The oxygen radical absorption capacity (ORAC) assay is based on antioxidant-mediated scavenging of peroxyl radical produced by thermal decomposition of 2,2′-azobis (2-amidino-propane) dihydrochloride (AAPH; Sigma-Aldrich, St. Louis, MO, USA) at 37 °C. Fluorescein was used as a fluorescent probe and protocol was modified according to Číž et al. [33]. The hemolymph stored with phenylthiourea at −80 °C was diluted 200× in PBS (pH 7.0) and 10 µL was transferred to the wells of a microplate. The samples were mixed with 170 µL of 100 nM fluorescein sodium salt (Sigma-Aldrich, St. Louis, MO, USA) and incubated for 10 min at 37 °C. After the incubation, 20 µL of 0.1 M AAPH was quickly added to the wells to start the reaction. Fluorescence (excitation 460 nm/emission 535 nm) was measured every 30 s for 2 h at 37 °C with shaking prior to each read using plate reader Sense (Hidex, Turku, Finland). Calibration prepared from gradually diluted 6-hydroxy-2,5,7,8-tetramethylchromane-2-carboxylic acid (Trolox; Sigma-Aldrich, St. Louis, MO, USA) and blank containing only PBS were measured together with the hemolymph samples. The ORAC results were analyzed as net area under the curve. The values corresponding to the hemolymph samples were compared to Trolox calibration and their antioxidant capacity expressed as mM equivalent of Trolox.

### 2.6. Determination of the Antimicrobial Activity

The antimicrobial activity was measured according to protocol described in Kunc et al. [30] as the ability of bumble bee hemolymph to inhibit growth of the Gram-positive bacteria *Micrococcus luteus* (CCM 169). Briefly, 5 µL of the hemolymph with phenylthiourea was applied into wells punctured in LB agar (4% agar in LB, MOBIO, Carlsbad, CA, USA) plates supplemented with bacteria *M. luteus* and incubated at 30 °C for 24 h. The resulting inhibition zones around the wells were measured and compared to calibration prepared from pure lysozyme (EC 3.2.1.17; Sigma-Aldrich, St. Louis, MO, USA). The antimicrobial activity in the hemolymph was expressed as the concentration of lysozyme with equal inhibitory effects.

### 2.7. Immunization of Bumble Bees

The antimicrobial response was induced in bumble bee workers from laboratory culture by injection of non-pathogenic bacteria. The 1:1 mix of heat-killed Gram-negative *Escherichia coli* (CCM 3954/ATCC 25922) and Gram-positive *Paenibacillus larvae* (CCM 4483) containing 10^5^ bacterial cells per 5 µL was injected into the abdomen between fourth and fifth tergal segment. The control group was injected with 5 µL of sterile phosphate buffer (pH 7.0). Prior to the injection, bumble bees were anesthetized with CO_2_ and their abdomen surface sterilized with 96% ethanol. The injected animals were left to recover for 24 h before their hemolymph was collected as mentioned above. We did not observe any significant mortality after injection.

### 2.8. Determination of the Phenoloxidase (PO) Activity

The phenoloxidase activity was modified according to previously published protocol [34]. The hemolymph of six bumble bees (2 µL of hemolymph per individual) was collected to ice-cold phosphate buffer (10 mM; pH 7.0) and pooled to obtain samples for the PO assay. The hemolymph was diluted 20× by phosphate buffer and immediately processed for the measurements. To prepare cell-free hemolymph plasma, the collected samples were snap frozen at −80 °C, melted on ice with occasional gentle mixing and centrifuged (15 min; 12,000× *g*; 4 °C). For measurement of constitutive PO activity, 95 µL of the supernatant was transferred to 96-well plate kept on ice, mixed with 5 µL of ultrapure water and 50 μL of substrate 3,4-dihydroxy-DL-phenylalanine (3 mg/mL in phosphate buffer; pH 7.0; Sigma-Aldrich, St. Louis, MO, USA). The increase of absorbance at 492 nm was measured at 30 °C by plate reader Sense (Hidex, Turku, Finland) for 30 min in one-minute intervals. The PO activity was determined as melanization rate per 30 min of reaction and expressed as net area under the curve. To determinate the total PO activity, an inactive proenzyme prophenoloxidase (pPO) present in cell-free plasma was activated by enzymatic cleavage prior to the measurement. The supernatant (95 µL) prepared as above was mixed with 5 µL of α-chymotrypsin (5 mg/mL in ultrapure water) and incubated 10 min at room temperature. After the incubation, 50 μL of 3,4-dihydroxy-DL-phenylalanine was added and measured as described above.

### 2.9. Statistical Analysis

Statistical analysis was performed in Prism 7.0 (GraphPad Software, San Diego, CA, USA). To assay normality and homogeneity of the data Shapiro–Wilk and Brown–Forsythe tests were used, respectively. For the statistical analysis of groups with normal distribution, one-way ANOVA with post-hoc Tukey’s test was used; data without normal distribution were tested with Kruskal–Wallis and post-hoc Dunn’s test. Statistical tests and number of replicates are specified in the figure legends. Differences were considered statistically significant for *p* values < 0.05. The detailed results of statistical analyses are summarized in the supplementary Tables S1 and S2.

## 3. Results

### 3.1. The Levels of Nutrients in the Hemolymph

**Figure 1:**
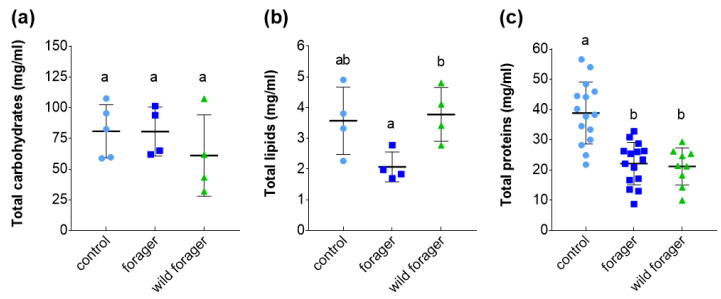
The total levels of nutrients in the hemolymph of non-foraging control, foraging and wild *Bombus terrestris*. The total concentration of (**a**) carbohydrates and (**b**) lipids were measured in the pooled hemolymph samples (*n* = 4–5; five animals per biological replicate which means at least 20 bumble bee workers per each group); (**c**) protein concentration was determined in individual animals (*n* = 9–15 bumble bee workers per group). Data presented as mean ± SD; significant differences *p* < 0.05 are indicated by different letters above the columns (Tukey’s test).

The results of the anthrone assay, which detects all essential carbohydrates including glucose and trehalose, as well as more complex saccharides, did not show any effect of foraging or laboratory conditions on carbohydrate levels in *B. terrestris* hemolymph (ANOVA summary F_2, 10_ = 0.8353, *p* = 0.4619; Figure 1a). However, the hemolymph of foraging bumble bees produced under laboratory conditions was shown to have a lower amount of lipids compared to that of wild bumble bees (forager vs. wild forager: *p* = 0.0482; Figure 1b and Table S1). The total lipid level of non-foraging bumble bees was the same as that of foragers produced in the laboratory and wild foragers (control vs. forager: *p* = 0.0812; control vs. wild forager: *p* = 0.9402; Table S1). The highest protein concentration was found in the non-foraging control bumble bees (Figure 1c). Both foraging groups, i.e., laboratory-reared foragers and wild foragers, showed significantly lower protein levels irrespective of their origin (control vs. forager: *p* < 0.0001; control vs. wild forager: *p* < 0.0001; forager vs. wild forager: *p* = 0.9614; Table S1).

### 3.2. Total Hemocyte Count

**Figure 2:**
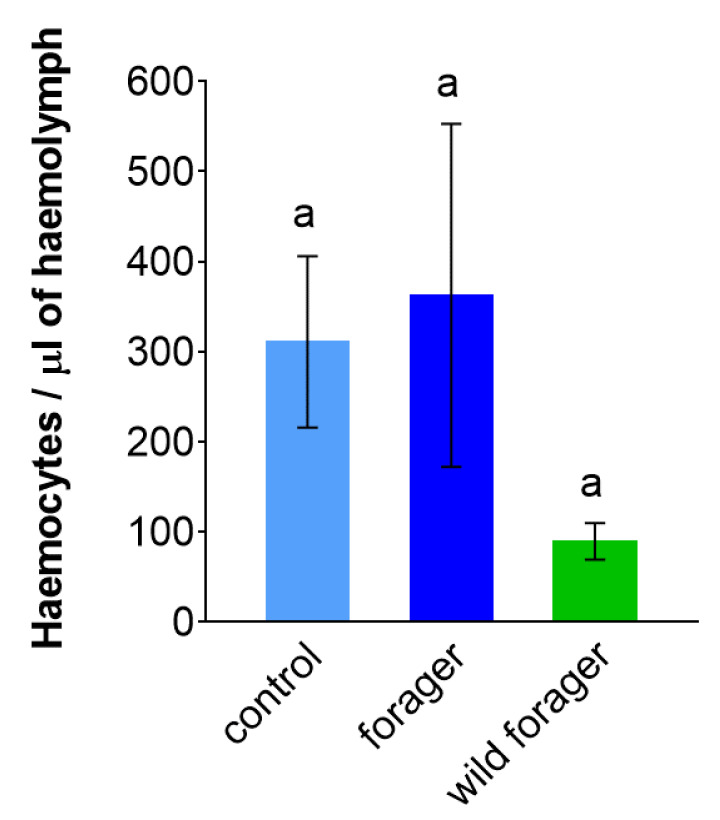
The concentration of circulating hemocytes determined in the hemolymph of non-foraging control, foraging and wild *Bombus terrestris* (*n* = 3–4; five animals per biological replicate which means at least 15 bumble bee workers per each group). Data presented as mean ± SD; significant differences *p* < 0.05 are indicated by different letters above the columns (Tukey’s test).

The number of circulating hemocytes observed in *B. terrestris* hemolymph showed high individual variability and did not differ among examined groups (Figure 2; Table S1). The lowest hemocyte concentration was found in wild foragers; however, this reduced concentration was not statistically significant when compared with the other groups (control vs. forager: *p* = 0.8439; control vs. wild forager: *p* = 0.1029; forager vs. wild forager: *p* = 0.0619; Table S1).

### 3.3. Antioxidant Capacity

**Figure 3:**
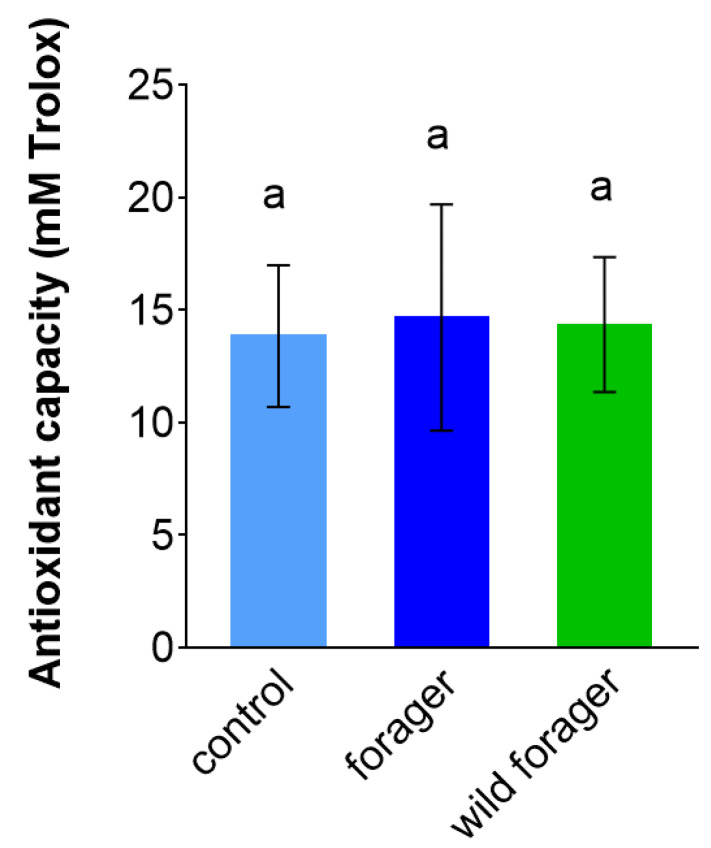
The antioxidant capacity determined in the pooled hemolymph samples from non-foraging control, foraging and wild *Bombus terrestris* workers (*n* = 3–4; five animals per biological replicate which means at least 15 bumble bee workers per each group). The concentration of antioxidants was quantified using the standard antioxidant Trolox. Data presented as mean ± SD; significant differences *p* < 0.05 are indicated by different letters above the columns (Dunn’s test).

The concentration of antioxidants measured by the ORAC was not shown to be affected by foraging activity or laboratory rearing conditions (ANOVA summary F_2, 7_ = 0.3364, *p* = 0.8952; Figure 3 and Table S1). In all three tested bumble bee groups, the mean values of antioxidant capacity were around 14 mM of the standard antioxidant Trolox.

### 3.4. Antimicrobial Activity

**Figure 4:**
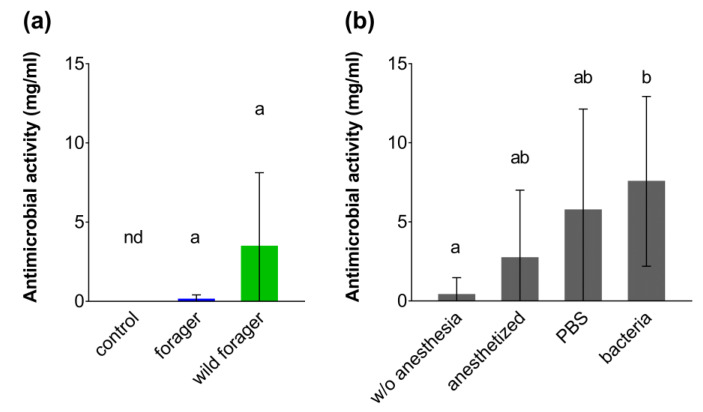
Antimicrobial activity in the *Bombus terrestris* hemolymph expressed as the concentration of lysozyme with equal antimicrobial effect. (**a**) Constitutive antimicrobial activity was measured in the pooled hemolymph samples from non-foraging control, foraging and wild bumble bees (*n* = 4; five animals per biological replicate which means 20 bumble bee workers per each group). (**b**) To induce the antimicrobial activity, the laboratory-reared bumble bee workers were immunized by injection of bacteria and compared to naïve individuals without CO_2_ anesthesia, CO_2_ anesthetized and phosphate buffer (PBS) injected bumble bees (*n* = 7–8; five animals per biological replicate which means at least 35 bumble bee workers per each group). Data presented as mean ± SD; nd = not detected activity; significant differences *p* < 0.05 are indicated by different letters above the columns (Dunn’s test).

Antimicrobial activity of *B. terrestris* hemolymph was measured by its ability to inhibit growth of the Gram-positive bacteria *Micrococcus luteus*. The hemolymph of control animals kept under laboratory conditions did not show any detectable antimicrobial activity, unlike that of foragers and wild foraging workers, which did exhibit low *M. luteus* growth-inhibiting activity (Figure 4a). In bumble bees reared in the laboratory, the injection of bacteria into the hemocoel induced an antimicrobial response and significantly increased the activity against *M. luteus* (w/o anesthesia vs. bacteria: *p* = 0.0122; Figure 4b and Table S1). Moderate induction of antimicrobial activity was observed in bumble bee workers 24 h after CO_2_ anesthesia and after phosphate buffer injection, however, this activity was not significantly higher than constitutive activity in the hemolymph of the control bumble bees (w/o anesthesia vs. anesthetized: *p* = 0.6844; w/o anesthesia vs. PBS: *p* = 0.1515; Table S1).

### 3.5. Phenoloxidase Activity

**Figure 5:**
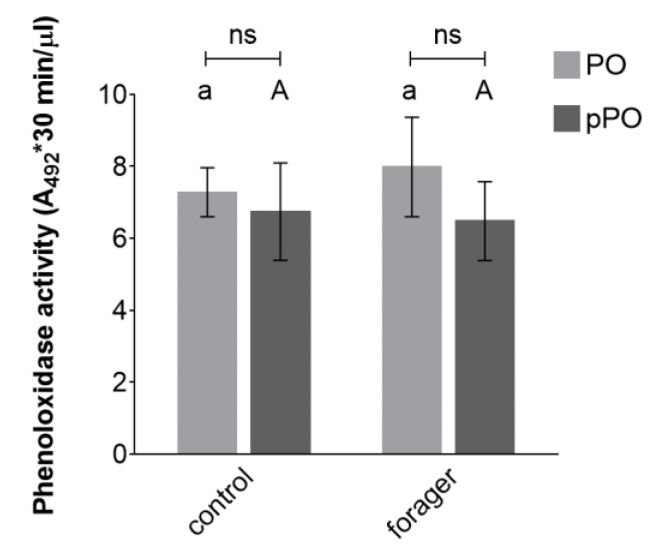
The constitutive phenoloxidase activity (PO) and total phenoloxidase activity measured after enzymatic cleavage of prophenoloxidase (pPO) in cell-free hemolymph of non-foraging control and foraging *Bombus terrestris* (*n* = 3; six animals per biological replicate which means 18 bumble bee workers per each group). The enzymatic reaction was measured for 30 min and PO activity expressed as integral of absorbance per µL of hemolymph. Data presented as mean ± SD; significant differences *p* < 0.05 between control and forager group are indicated by different small and capital letters above the columns for PO and pPO, respectively (*t*-test); ns = not significant difference between PO and pPO assay (*t*-test).

Both constitutive and total phenoloxidase activity were determined in the hemolymph plasma of laboratory-reared *B. terrestris* workers, and different according to their foraging activity (Figure 5). Due to the high demands of our methodology on the volume of hemolymph required for analysis, enzymatic activity was not determined in wild foraging bumble bees that were not available in sufficient numbers. The constitutive and total phenoloxidase activity of non-foraging bumble bees was fully comparable to the activity observed in foraging individuals (*p* = 0.4759 and *p* = 0.8102 for constitutive and total activity, respectively; Figure 5 and Table S2). Interestingly, the total PO activity measured after proteolytic activation of pPO did not differ from the constitutive PO activity found in the same bumble bees (*p* = 0.5707 and *p* = 0.2158 for control and foragers, respectively; Table S2).

## 4. Discussion

To ensure high production rates and welfare standards, animal rearing conditions are generally optimized for kept species; this also applies to the production of bumble bees. Although this approach is ideal for commercial production, it is far from the natural situation, and therefore might reduce the success rate of laboratory-reared animals when exposed to field conditions. Specifically, bumble bees reared in captivity are used to ad libitum feeding, restricted flight activity and lack of immune stimuli, i.e., generally low energy expenditure; whilst wild individuals are forced to spend a considerable amount of energy on all of these activities. The costs of flight and trade-off between immunity and other physiological systems of insects are well known [14,20,22,27,35,36].

Measuring total nutrient levels enabled us to monitor metabolic changes that accompany the transition of nest-inhabiting *B. terrestris* workers to foragers. Unsurprisingly, the altered activity, caused by the change in lifestyle of bumble bees, affected nutrients in the hemolymph, however, foraging did not change the carbohydrate and protein levels of bumble bees reared in the laboratory to levels higher or lower than what was observed in the wild animals (Figure 1).

Carbohydrates are the main source of energy for flight and therefore are crucial for bee workers, especially the foraging individuals [7,9,37,38,39], which play a key role in colony economy and reproduction. Due to flight muscle activity, hemolymph carbohydrates can be rapidly exhausted, but, under normal circumstances, can be replenished into the circulation by utilization of storage saccharides or other nutrients [27]. We assume that the rapid nutrient turnover could be the cause of the stable carbohydrate level in the hemolymph that we observed in all experimental groups. Feeding bees have been shown to prefer intake of carbohydrates over other nutrients [5,9], and foragers of eusocial insects are known to have higher saccharide demands than other members of the colony [9], which further supports the need for tight regulation of carbohydrate intake and maintenance of a constant level of carbohydrates in the hemolymph of active individuals.

Unlike carbohydrates, the total lipid level in the hemolymph decreased in laboratory-reared bumble bees after their application in the field, which was most likely a consequence of increased activity or a change in food availability and quality for foragers. In honey bees, an abundance of stored lipids was found to negatively correlate with foraging, as the nest-inhabiting workers depleted their lipid reserves with the onset of foraging [40].

It is worthy of note that in our study a decrease in lipid level was only observed in the circulation of laboratory-reared foragers, while that of the wild foragers was comparable to the non-foraging control animals (Figure 1b). It was not feasible to ascertain whether the sampled wild workers were of the same age, or came from colonies with similar nutritional needs, as bees of the experimental nests produced under the laboratory conditions. However, both of these factors can influence the spectrum of nutrients collected, metabolized and brought to the nest by foragers [8,11,37]. For bees, the only source of lipids is pollen [7,8], the availability of which depends on a diverse distribution of floral resources in the vicinity of the colony. However, in our study the wild bumble bees were collected from a locality similar to where we applied the laboratory-reared foragers; therefore, we assume that the observed difference in the lipid level of foragers was not due to the availability and quality of food sources.

Lipids are essential for the development of both bee larvae and adults [7], and also provide a large store of energy which is utilized to replenish saccharide levels during longer flight or starvation episodes [27]. It can be speculated that the low level of hemolymph lipids observed in the laboratory-reared foragers results from high flight activity rather than increased demand on nutrients for colony development, since we did not observe any notable differences in colony growth between non-foragers and foragers (data not shown). In regards to field application, deficiency of specific lipids is a risk factor which has been proven to have a significant impact on behavioral development and learning in honey bees [41,42].

Foraging is a physically demanding activity that increases metabolism of all nutrients, including proteins. Accordingly, we observed a concentration of hemolymph proteins in all foraging groups that was almost twice as low as that of the non-foraging bumble bees. The previous studies described higher demands for essential amino acids and proteins in larvae and nursing honey bees than for foraging individuals, which require high carbohydrate intake [5,9]. Although bumble bees have been shown to collect pollen with higher protein quality than honey bees [43], and to discriminate between food sources based on nutrient content [44], the workers seem to forage for pollen primarily to supply protein stores in-colony [45], rather than to replenish amino acids at the individual level.

It is important to note that both protein deficiency and an excess of protein or amino acids can negatively impact bumble bee health. In *B. terrestris*, protein-starved workers have been shown to have an impaired immune response to natural parasites [46], and reduced learning ability when immune-challenged, even with non-pathogenic stimuli [47]. On the other hand, bumble bee workers fed with high protein and free essential amino acids diets suffer from higher mortality [5], and reduced life span has also been observed in honey bees [48]. All colonies used in our study grew and developed alike (based on numbers of colony members), therefore, we infer that neither the increased hemolymph protein level in non-foraging workers nor the decreased protein level in foragers has a significant impact on bumble bee performance.

Nevertheless, the changes in nutrients at the hemolymph level confirm that diet composition, with the particular emphasis on quality of proteins, is the critical factor for bumble bees [49]. Availability of food sources affects many aspects of bumble bee biology and ecology, and therefore must be taken into account when rearing bumble bees in a laboratory or applying them in the field.

The connection between nutritional intake and immune response is well established in bumble bees; even their reaction to non-pathogenic stimuli causes a significant increase in energy consumption [35]. Higher antimicrobial activity observed in the wild bumble bees is typically attributed to natural immunization by microorganisms and other pathogenic, as well as non-pathogenic, stimuli present in the environment [50,51]. Unlike the wild individuals or foraging workers, the non-foraging bumble bees can be considered to have been reared under aseptic conditions with a low level of immune elicitors and a lack of pathogens. Accordingly, we did not detect any antibacterial activity against Gram-positive bacteria in the hemolymph of naïve bumble bees, whereas the injection of heat-killed bacteria induced an immune response and led to the observed increase in antimicrobial activity, which was comparable to that observed in wild bumble bees.

Although the genome of eusocial insects is typically characterized by a reduced number of immune genes [52], a broad spectrum of inducible antimicrobial peptides has been described in bumble bees and honey bees including, for instance, abaecin, apidaecin, hymenoptaecin, defensin and lysozyme [53,54,55]. Together with other antimicrobial agents, such as cytotoxic molecules formed during activation of the PO system, or reactive oxygen species produced in some insect species by phagocytic cells, antimicrobial peptides can provide effective protection against bacterial and fungal infections [19,22,56]. Exposure to carbon dioxide has been shown to promote an immune response against injected bacteria in *B. impatiens* [57]. In line with the previous research, our results confirm an effect of carbon dioxide narcosis on the humoral branch of the bumble bee immunity, specifically an increase in antimicrobial activity 24 h after CO_2_ exposure.

The contribution of PO activity to the humoral immune response was measured as the rate of PO-catalyzed melanization. Melanization has been shown to negatively correlate with age in *B. terrestris* and *B. lucorum* reared under laboratory conditions [4,58], as well as in wild populations of *B. muscorum* [59]. When comparing non-foraging bumble bees with foragers, we did not observe any differences in constitutive or total PO activity, suggesting that foraging does not influence the activation of the PO system nor the synthesis of its components. This finding is in accordance with previous research on *B. terrestris* which found PO activity to be independent of type and nutritional value of pollen diet [36]. Since pollen is the major source of proteins and lipids, it has been suggested that PO activity depends on nectar availability and carbohydrate intake instead [60,61]. Alas, no changes in carbohydrate level or saccharide intake that could be correlated to changes of PO activity were observed in our study, nor in previous research, to confirm this hypothesis. However, a strong negative correlation of melanization response with an abundance of parasites was observed in wild bumble bees *B. muscorum* after an immune challenge with a nylon implant [59]. This previous finding suggests that a combination of multiple negative factors, such as dietary restrictions and high parasite prevalence, reduces immunocompetence in wild bumble bees, whereas simple disturbances can be compensated for.

Contrary to our expectations, the constitutive PO activity was equal to the total PO activity found in the same hemolymph samples, which suggests that hemolymph collection, freezing, thawing, and other manipulations, lead to overall activation of the PO system (for further discussion see [62]). In future studies, the constitutive PO activity should be preferentially measured in whole hemolymph immediately after its collection, and several factors that negatively interfere with this assay (i.e., significant temperature changes and excessive handling of collected hemolymph) must be avoided to prevent unintentional activation of the PO system.

Similar to PO activity, we did not observe any changes that could be related to foraging in the level of antioxidants. These compounds participate in scavenging of potentially harmful cytotoxic molecules (e.g., free radicals) that are produced, for instance, during phagocytosis or activation of the PO system [63]. Antioxidants form a diverse group of chemical compounds and enzymes [64] that differ in specificity and mode of action, but act in a coordinated way. We used the ORAC assay to determine the total antioxidant capacity of bumble bee hemolymph.

Since bees feed on floral resources that can be rich in potent antioxidants, such as flavonoids and other phenolic substances [65,66], we expected to observe a difference in antioxidant capacity when comparing the wild foragers to non-foraging bee workers kept under laboratory conditions. Surprisingly, foraging did not affect antioxidant activity. It can be speculated that all bumble bee groups were able to keep the level of antioxidants at a maximum, since both the laboratory diet and floral sources in summer (i.e., when this study was carried out) provide sufficient supply of potential antioxidants. Antioxidant activity in bees also varies with age and health condition [67,68], however, the impact of both of these factors was minimized in our study by using workers of approximately the same age and without visible behavioral or visual signs of infection.

The total concentration of hemocytes in the hemolymph of *B. terrestris* has been proven to be independent of the age of the workers [4]. In our experiments, the number of hemocytes circulating in the hemolymph of foraging, non-foraging and wild bumble bee workers was comparable, even after two weeks of foraging. This observation is in line with the previous study of Korner and Schmid-Hempel [62], which found that hemocyte counts in the hemolymph of *B. terrestris* were constant over 14 days of conducted study, except for temporary several-hour fluctuations caused by an immune challenge.

Nevertheless, the manifestation of cellular response is not simply a matter of hemocyte concentration, but also the hemocytic activity, as confirmed by König and Schmid-Hempel [14]. The present study observed a significant decrease in encapsulation in the foraging *B. terrestris* workers compared to non-foraging bumble bees. The hemocyte-mediated encapsulation response to implanted nylon was impaired after 13 h of foraging activity. Further studies that include the identification of specific hemocytic populations and determine their translational activity could help characterize the nature of the decreased cellular immunocompetence observed in bee foragers [14,15,58].

## 5. Conclusions

The present study shows that active foraging is reflected both at the level of nutrients in hemolymph and immunity; however, the changes observed herein in bumble bee workers reared in the laboratory were not markedly different than the physiological and immunological status of collected wild bumble bees. Two weeks of foraging activity had a negligible effect on total carbohydrate and antioxidant levels in the hemolymph, as well as on the concentration of hemocytes mediating cellular immune response and phenoloxidase activity responsible for melanization. On the other hand, the total protein and lipid concentrations decreased after foraging in laboratory-reared bumble bees, which confirmed our assumption of high energy demands of foraging activity. Although the physiological levels of nutrients were affected by the foraging, both wild and laboratory-reared bumble bee workers were found to be equally immunocompetent and prepared for field application. The supposed trade-off between foraging activity and immunity is not general, as proved in the laboratory-reared foragers that showed a higher antimicrobial response than the non-foragers. We suggest that this induction of antimicrobial activity can be attributed to a natural immunization by microorganisms and antigens present in the environment, because the same effect was observed in *B. terrestris* after the experimental in vivo immune challenge. The physiological and immunological response observed in the foraging *B. terrestris* workers provides further evidence confirming the ability of laboratory-reared bumble bees to adapt to the field conditions comparably to the wild bumble bees.

## Supplementary Materials

The following are available online at https://www.mdpi.com/2075-4450/11/5/321/s1, Table S1: Summary of statistical analyses. The data with normal distribution (total concentration of carbohydrates, lipids, proteins and hemocytes) were analysed using one-way ANOVA with post-hoc Tukey’s test, whereas Kruskal-Wallis with post-hoc Dunn’s test was used to analyse data without normal distribution (antioxidant activity, constitutive and induced antimicrobial activity). Significant *p* values < 0.05 are highlighted in bold. * F or H statistic is reported for ANOVA and Kruskal-Wallis test, respectively. Table S2: Summary of t tests that were used to analyse phenoloxidase activity in the hemolymph of *Bombus terrestris* workers. The constitutive phenoloxidase activity (PO) and total phenoloxidase activity measured after enzymatic cleavage of prophenoloxidase (pPO) were analysed in control and foraging *B. terrestris*.
