# Supplementary material for: The Effect of Foraging on Bumble Bees, Bombus terrestris, Reared under Laboratory Conditions
Source: Insects. 2020 May 23;11(5):321. doi: 10.3390/insects11050321 (PMC7290516; doi:10.3390/insects11050321)
Supplement: Supplementary file 1 [file insects-11-00321-s001.zip › insects-753051-suppl/Table S1.docx]

**Table S1.** Summary of statistical analyses. The data with normal distribution (total concentration of carbohydrates, lipids, proteins and hemocytes) were analysed using one-way ANOVA with post-hoc Tukey's test, whereas Kruskal-Wallis with post-hoc Dunn’s test was used to analyse data without normal distribution (antioxidant activity, constitutive and induced antimicrobial activity). Significant *p* values < 0.05 are highlighted in bold. * F or H statistic is reported for ANOVA and Kruskal-Wallis test, respectively.

| **Tested Parameter** | **ANOVA Summary** | **Sum of Squares** | **Df** | **Mean Square** | **F/H *** | ***p*-Value** | **Post-Hoc Comparisons** | ***p*-Value** |
| --- | --- | --- | --- | --- | --- | --- | --- | --- |
| Carbohydrates | Treatment (between columns) | 1062 | 2 | 531.1 | 0.8353 | 0.4619 | control vs. forager | >0.9999 |
|  | Residual (within columns) | 6358 | 10 | 635.8 |  |  | control vs. wild forager | 0.4997 |
|  | Total | 7421 | 12 |  |  |  | forager vs. wild forager | 0.5403 |
| Lipids | Treatment (between columns) | 6.934 | 2 | 3.467 | 4.73 | **0.0395** | control vs. forager | 0.0812 |
|  | Residual (within columns) | 6.597 | 9 | 0.733 |  |  | control vs. wild forager | 0.9402 |
|  | Total | 13.53 | 11 |  |  |  | forager vs. wild forager | **0.0482** |
| Proteins | Treatment (between columns) | 2699 | 2 | 1350 | 19.68 | **<0.0001** | control vs. forager | **<0.0001** |
|  | Residual (within columns) | 2469 | 36 | 68.58 |  |  | control vs. wild forager | **<0.0001** |
|  | Total | 5168 | 38 |  |  |  | forager vs. wild forager | 0.9614 |
| Hemocytes | Treatment (between columns) | 129062 | 2 | 64531 | 4.493 | 0.0556 | control vs. forager | 0.8439 |
|  | Residual (within columns) | 100536 | 7 | 14362 |  |  | control vs. wild forager | 0.1029 |
|  | Total | 229598 | 9 |  |  |  | forager vs. wild forager | 0.0619 |
| Antioxidant capacity | Treatment (between columns) |  | 2 |  | 0.3364 | 0.8952 | control vs. forager | >0.9999 |
|  | Residual (within columns) |  | 7 |  |  |  | control vs. wild forager | >0.9999 |
|  | Total |  | 9 |  |  |  | forager vs. wild forager | >0.9999 |
| Constitutive antimicrobial activity | Treatment (between columns) |  | 2 |  | 2.399 | 0.3697 | control vs. forager | >0.9999 |
|  | Residual (within columns) |  | 8 |  |  |  | control vs. wild forager | 0.3960 |
|  | Total |  | 10 |  |  |  | forager vs. wild forager | 0.9277 |
| Induced antimicrobial activity | Treatment (between columns) |  | 3 |  | 10.28 | 0.0163 | w/o anesthesia vs. anesthetized | 0.6844 |
|  |  |  |  |  |  |  | w/o anesthesia vs. PBS | 0.1515 |
|  | Residual (within columns) |  | 27 |  |  |  | w/o anesthesia vs. bacteria | **0.0122** |
|  |  |  |  |  |  |  | anesthetized vs. PBS | >0.9999 |
|  | Total |  | 30 |  |  |  | anesthetized vs. bacteria | 0.7161 |
|  |  |  |  |  |  |  | PBS vs. bacteria | >0.9999 |
