# Supplementary material for: The Effect of Foraging on Bumble Bees, Bombus terrestris, Reared under Laboratory Conditions
Source: Insects. 2020 May 23;11(5):321. doi: 10.3390/insects11050321 (PMC7290516; doi:10.3390/insects11050321)
Supplement: Supplementary file 1 [file insects-11-00321-s001.zip › insects-753051-suppl/Table S2.docx]

**Table 2.** Summary of t tests that were used to analyse phenoloxidase activity in the hemolymph of *Bombus terrestris* workers. The constitutive phenoloxidase activity (PO) and total phenoloxidase activity measured after enzymatic cleavage of prophenoloxidase (pPO) were analysed in control and foraging *B. terrestris*.

| **Tested Parameter** | ***t*-Test** | | **Dfn** | **Dfd** | **F** | ***p*-Value** |
| --- | --- | --- | --- | --- | --- | --- |
| Phenoloxidase activity | control vs. forager | PO | 2 | 2 | 4.122 | 0.4759 |
|  |  | pPO | 2 | 2 | 1.520 | 0.8102 |
|  | PO vs. pPO | control | 2 | 2 | 3.955 | 0.5707 |
|  |  | forager | 2 | 2 | 1.585 | 0.2158 |
